# Breathomics to monitor interstitial lung disease associated with systemic sclerosis

**DOI:** 10.1183/23120541.00175-2024

**Published:** 2024-09-16

**Authors:** Thibault Massenet, Judith Potjewijd, Rachid Tobal, Fanny Gester, Delphine Zanella, Monique Henket, Makon-Sébastien Njock, Thibaut Dejong, Gregory Gridelet, Laurie Giltay, Françoise Guissard, Béatrice André, Clio Ribbens, Renaud Louis, Pieter Van Paassen, Julien Guiot, Pierre-Hugues Stefanuto

**Affiliations:** 1 Molecular System, Organic & Biological Analytical Chemistry Group, University of Liège, Liege, Belgium; 2 Department of Internal Medicine, Division of Clinical and Experimental Immunology, Maastricht University Medical Center, Maastricht, The Netherlands; 3 Respiratory Medicine, CHU Liège, Liege, Belgium; 4 Rheumatology Department, CHU Liège, Liege, Belgium; 5 J. Guiot and P-H. Stefanuto contributed equally as senior authors

## Abstract

Systemic sclerosis (SSc) is an autoimmune disease of unknown origin characterised by an inflammatory process associated with vascular damage and collagen deposition. Interstitial lung disease (ILD) is highly prevalent in SSc (SSc-ILD), is known to be the leading cause of death among these patients and its treatment requires aggressive multimodal therapy [1]. In this context, there is a major clinical need to identify significant SSc-ILD at the earliest stage, especially for patients at risk of developing a progressive form of the disease. Nowadays, few biomarkers can classify patients at risk of developing SSc-ILD; most of them are blood-based and detected in the last clinical stage of the disease. Previously, we have demonstrated that SSc patients exhibit a specific signature of volatile organic compounds (VOCs) compared to healthy subjects [2]. In this prospective study, we aimed to identify the potential of VOCs to predict the ILD phenotype.

## Shareable abstract

**Exhaled breath analysis, utilising VOCs, offers promise in identifying SSc-ILD patients, aiding targeted treatment. Further multicentric studies are crucial for validation and exploring longitudinal VOC changes for comprehensive disease management.** https://bit.ly/4aKMYif

*To the Editor:*

Systemic sclerosis (SSc) is an autoimmune disease of unknown origin characterised by an inflammatory process associated with vascular damage and collagen deposition. Interstitial lung disease (ILD) is highly prevalent in SSc (SSc-ILD), is known to be the leading cause of death among these patients and its treatment requires aggressive multimodal therapy [1]. In this context, there is a major clinical need to identify significant SSc-ILD at the earliest stage, especially for patients at risk of developing a progressive form of the disease. Nowadays, few biomarkers can classify patients at risk of developing SSc-ILD; most of them are blood-based and detected in the last clinical stage of the disease. Previously, we have demonstrated that SSc patients exhibit a specific signature of volatile organic compounds (VOCs) compared to healthy subjects [2]. In this prospective study, we aimed to identify the potential of VOCs to predict the ILD phenotype.

The study presented was conducted on a cohort composed of 42 patients, 21 patients suffering from SSc and 21 suffering from SSc-ILD. These patients were prospectively recruited both in University Hospital of Liège (CHU), Belgium, and Maastricht University Medical Center (MUMC+), the Netherlands, during a period of 6 months starting in July 2021 and ending in September 2021. SSc was diagnosed according to 2013 American College of Rheumatology/European League Against Rheumatism guideline [3]. SSc-ILD was defined by a specific interstitial lung involvement confirmed through a multidisciplinary discussion as recommended by American Thoracic Society/European Respiratory Society guidelines [4]. The protocol was locally approved by ethical committees (Belgian number B707201422832, reference Liege 2014/302; Dutch number NL57351.068.17, reference Maastricht 172021). All subjects gave written informed consent before participating to the study. All breath samples were systematically collected in the same room at the two medical facilities to minimise the effect of variation in background air. As established in our standard operating procedures (SOPs), breath sampling was conducted before any pulmonary function test and patients were not required to fast [5]. The exhaled breath samples were collected in inert 5-L Tedlar bags. The content of the sampling bag was subsequently concentrated under standardised conditions into Tenax GR/Carbopack B TD tubes (Markes International Ltd). Following the collection process, the tubes were hermetically sealed using specific caps for preservation before being analysed. The exhaled air was finally analysed by thermal desorption comprehensive two-dimensional gas chromatography high-resolution time-of-flight mass spectrometry (TD-GC×GC-HRMS) (Leco Corporation) at the OBiAChem laboratory in Belgium [2]. Statistical analyses were performed using RStudio (2022.12.0) and MetaboAnalyst online 5.0 [6]. For more detailed technical information, see previous research conducted [2, 5].

A total of 42 patients were recruited from two expert centres. The patients’ characteristics are presented in figure 1a.

**FIGURE 1:**
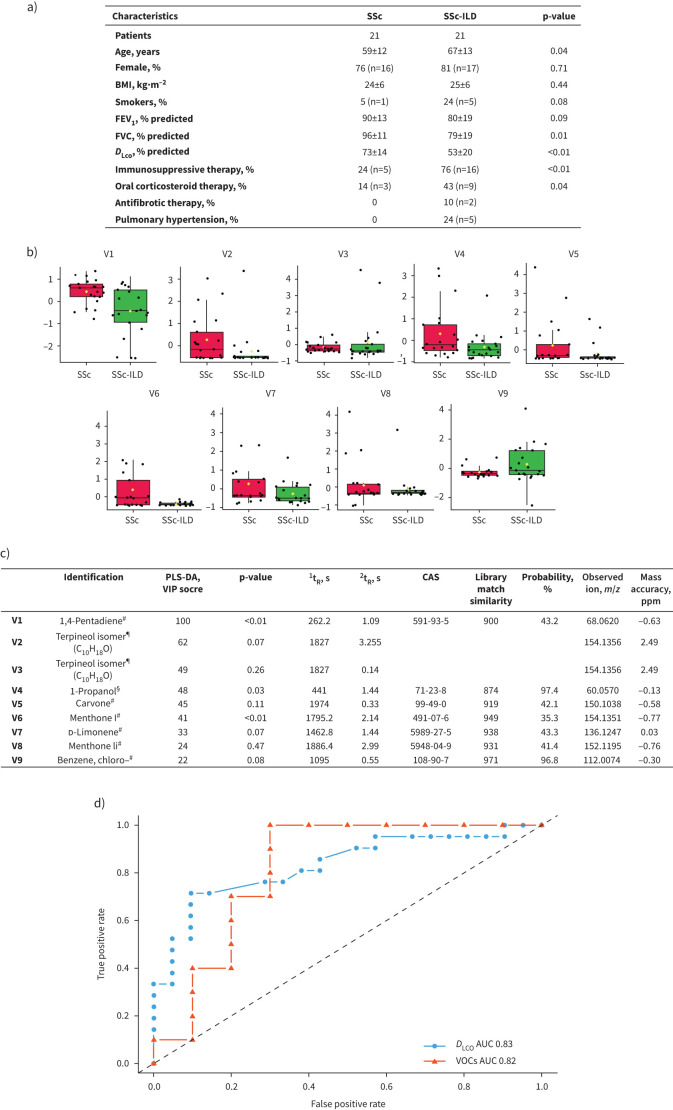
a) Patients’ characteristics. Data are presented as mean±sd unless otherwise stated. p-values were obtained through Wilcoxon–Mann–Whitney rank-sum test for continuous variables and Chi-squared test for noncontinuous variables. b) Boxplot of the nine metabolites identified in exhaled breath used to discriminate systemic sclerosis (SSc) patients (red) from SSc patients with interstitial lung disease (ILD) (green). The lower middle, and upper lines of the box represent the 25th, 50th and 75th percentiles. The upper and lower whiskers extend to 1.5 times the interquartile range. The numbering refers to the identification of the metabolites reported in c. From these nine compounds, we specifically identified eight compounds that were exhaled more in the breath of SSc patients compared to SSc-ILD. c) Table of the potential markers discriminating the two classes of patients. Mass spectral information (library match, probability and characteristic ion), partial least squares-discriminant analysis (PLS-DA) variable importance (VIP) score and p-value obtained after Wilcoxon rank-sum test are reported. The mass accuracy was calculated on the specified ion. Those specific compounds belong to the alkadiene (V1), terpenoid (V2, V3 and V5–V8) and alcohol (V4) chemical families. Conversely, we observed a reduction of chlorobenzene in SSc patients’ breath compared to SSc-ILD. d) Classification performances of the most influential metabolites identified in exhaled breath for SSc diagnosis compared to SSc-ILD diagnosis using a receiver operating characteristic (ROC) curve analysis based on PLS-DA algorithm (orange). Classification performances of diffusing capacity of the lung for carbon monoxide (*D*_LCO_) using ROC curve analysis based on univariate *D*_LCO_ analysis and a threshold value set at 61 (blue). BMI: body mass index; FEV_1_: forced expiratory volume in 1 s; FVC: forced vital capacity; ^1^*t*_R_: first-dimension retention time; ^2^*t*_R_: second-dimension retention time; CAS: Chemical Abstract Service; *m*: mass; *z*: charge; AUC: area under the curve; VOC: volatile organic compound. ^#^: Metabolomics Standards Initiative (MSI) level 2; ^¶^: MSI level 3; ^§^: MSI level 1.

In our study, we compared the exhaled breath composition between SSc and SSc-ILD patients using TD-GC×GC-HRMS. This technique allowed us to detect ∼800 features. We developed a statistical model based on partial least squares-discriminant analysis. This model was then subsequently employed to select nine significant markers based on their variable importance score (figure 1b and c). This model achieved an area under the curve (AUC) of 0.82, accuracy of 85%, sensitivity of 77% and specificity of 100% (figure 1d) for identifying the ILD phenotype. Furthermore, the achieved metrics were similar to the diffusing capacity of the lung for carbon monoxide (*D*_LCO_)-based univariate model, which achieved an AUC of 0.83 (figure 1d).

To evaluate robustness, we tested potential confounding factors such as smoking habits, treatments and gender, which were included in the metadata (figure 1a). We did not identify any interference in the predictive ability of VOCs by potential confounders. A correlation was observed between the functional respiratory parameters (*i.e. D*_LCO_ and forced vital capacity % predicted value) and the VOCs. A positive correlation was observed between *D*_LCO_ and the probability of classification of the VOC-based model.

We have identified a breath-based model able to discriminate SSc-ILD with a high sensitivity, confirming its potential in patient management. Four markers are in line with our previous study, reaffirming the potential of VOCs in disease classification (*i.e.* two terpineol isomers, menthone I and menthone II) with their potential metabolic pathways discussed in our earlier work [2]. A key focus of this research lies in the discovery of nine VOCs present in the exhaled breath of patients that exhibit the capability to discriminate between SSc and SSc-ILD. These nine markers demonstrated significant classification performance in comparison to conventional lung physiological markers and functional parameters [7]. Furthermore, we validated methodological SOPs to conduct breath-based multicentric studies, a determinant step toward validating our classification across several clinical centres. Multicentric breath studies are a major improvement for this emerging monitoring strategy.

Moreover, this finding contributes to an enhanced understanding of the disease and the associated metabolic pathways. For instance, 1,4-pentadiene, a hydrocarbon, emerges as a potential biomarker of several lung pathologies. We previously demonstrated that chemically and biologically induced inflammation in lung epithelial cells can lead to increased hydrocarbons levels due to inflammation-associated oxidative stress [8]. Another compound, 1-propanol, has been proposed as a potential marker for lung cancer, detected in the breath of cancer patients and in the headspace of cancer cells [9]. The presence of this alcohol might stem from the cytochrome P450 enzymes that hydroxylate lipid peroxidation biomarkers, generating alcohols. Notably, this last marker has also been observed in the exhaled breath of asthmatic patients and has discriminatory capabilities, along with other VOCs, in distinguishing between neutrophilic and eosinophilic asthma [5].

The constant exposure of humans to exogenous compounds through various sources such as diet and environment can lead to the direct secretion of these volatile compounds in breath. Additionally, volatile downstream products stemming from these compounds could potentially serve as medical probes [10]. Limonene (d-limonene), another terpene regarded as an exogenous marker, was found to be elevated in the breath of patients with liver cirrhosis [11]. Following entry into the bloodstream, limonene is metabolised by the P450 enzymes CYP2C9 and CYP2C19. This represents the second instance in this study where cytochrome P450 enzymes play a role. Conversely, carvone and chlorobenzene have yet to be associated with disease markers based on current knowledge. Like limonene, these compounds could be considered as a probe that assesses metabolism performances. It is worth noting that an increased amount of altered extracellular matrix components destroys alveolar architecture and disrupts gas exchange equilibrium [12]. Therefore, elevated volatile concentrations could be also attributed to the thickening of alveolar walls and subsequent impairment of gas exchange, influencing concentrations.

The accurate and sensitive statistical model presented in this study showed the potential of VOCs in exhaled breath to identify SSc-ILD patients in a SSc cohort. In addition, our study is corroborating the potential of four terpenes to discriminate SSc patients. Exhaled breath could help clinicians to rapidly provide targeted treatment to patients suffering from ILD. Nevertheless, prospective multicentric studies to further validate the potential of exhaled breath analysis for the management of SSc-ILD patients would be needed. Future studies would include SSc-ILD at early stages to evaluate longitudinal changes of VOCs compared to disease progression and treatment response.
